# Antimicrobial, Antioxidant and Antiproliferative Secondary Metabolites from *Inonotus nidus-pici*

**DOI:** 10.3390/molecules26185453

**Published:** 2021-09-07

**Authors:** Zsófia Garádi, Miklós Dékány, Ágnes M. Móricz, Anikó Gaál, Viktor Papp, Szabolcs Béni, Attila Ványolós

**Affiliations:** 1 Department of Pharmacognosy, Semmelweis University, Üllői út. 26, H-1085 Budapest, Hungary; garadi.zsofia@pharma.semmelweis-univ.hu (Z.G.); beni.szabolcs@pharma.semmelweis-univ.hu (S.B.); 2 Directorate of Drug Substance Development, Egis Pharmaceuticals Plc, P.O. Box 100, H-1475 Budapest, Hungary; 3 Spectroscopic Research, Gedeon Richter Plc., Gyömrői út 19-21, H-1103 Budapest, Hungary; m.dekany@richter.hu; 4 Plant Protection Institute, Centre for Agricultural Research, ELKH, Herman Ottó út 15, H-1022 Budapest, Hungary; moricz.agnes@atk.hu; 5 Biological Nanochemistry Research Group, Institute of Materials and Environmental Chemistry, Research Centre for Natural Sciences, Magyar Tudósok Körútja 2, H-1117 Budapest, Hungary; gaal.aniko@ttk.hu; 6 Department of Botany, Hungarian University of Agriculture and Life Sciences, Villányi út 29-43, H-1118 Budapest, Hungary; papp.viktor@uni-mate.hu

**Keywords:** *Hymenochaetaceae*, chaga, sclerotium, steroids, ceramide, antimicrobial, antiproliferative, doxorubicin resistant

## Abstract

*Inonotus nidus-pici* is a sterile conk which produces macrofungus, a neglected Central-Eastern European relative of the prized *Inonotus obliquus*, also known as chaga. Investigation of the methanol extract of the poroid fungus *I. nidus-pici* resulted in the isolation of citropremide (**1**), 3,4-dihydroxybenzalacetone (**2**) , lanosterol (**3**), ergost-6,8,22-trien-3β-ol (**4**), and ergosterol peroxide (**5**). The structures of fungal compounds were determined on the basis of one- and two-dimensional NMR and MS spectroscopic analysis. Compounds **1**–**2** and **4**–**5** were evaluated for their antioxidant and antimicrobial properties against several bacterial and fungal strains. 3,4-dihydroxybenzalacetone (**2**) and ergost-6,8,22-trien-3β-ol (**4**) demonstrated moderate antimicrobial activity, while the former possessed notable antioxidant activity in DPPH assay. The antiproliferative examinations performed on three human cancer (MES-SA, MES-SA/Dx5, A431) cell lines demonstrated that compounds **4** and **5** have notable cytotoxic activity with IC values in micromolar range. The current study represents the first report on the chemical profile of *I. nidus-pici*, providing a comprehensive study on the isolation and structure determination of bioactive secondary metabolites of this macrofungus.

## 1. Introduction

Higher macrofungi, also known as mushrooms, have been used as a source of food and for their medicinal (anticancer, anti-inflammatory, and immunomodulatory, etc.) properties since ancient times, particularly in the traditional medicines of the Far East. Recent mycochemical studies unequivocally demonstrate that mushrooms represent an almost unlimited reservoir of biologically active natural products with a high structural diversity: aromatic cadinane sesquiterpenoids from *Phellinus pini* with potential SARS-CoV-2 inhibitory activity [1], meroterpenoids from *Albatrellus yasudae* with amyloid-β aggregation inhibitory properties [2], and antibacterial diterpenoids from *Psathyrella candolleana* [3], among others. Fungal sclerotia are also notable sources of biologically active secondary metabolites, and many of these fungal structures are considered as prospective medicines or as a food source [4,5,6]. For instance, the sclerotia of some edible mushroom species (e.g., *Laccocephalum mylittae*, *Pleurotus tuber-regium*) are known as important food components, while others (e.g., *Lignosus* spp., *Pachyma* spp., *Polyporus umbellatus*) are utilized by traditional medicine and consumed principally for their positive healing effects [7,8,9,10]). 

**Figure 1:**
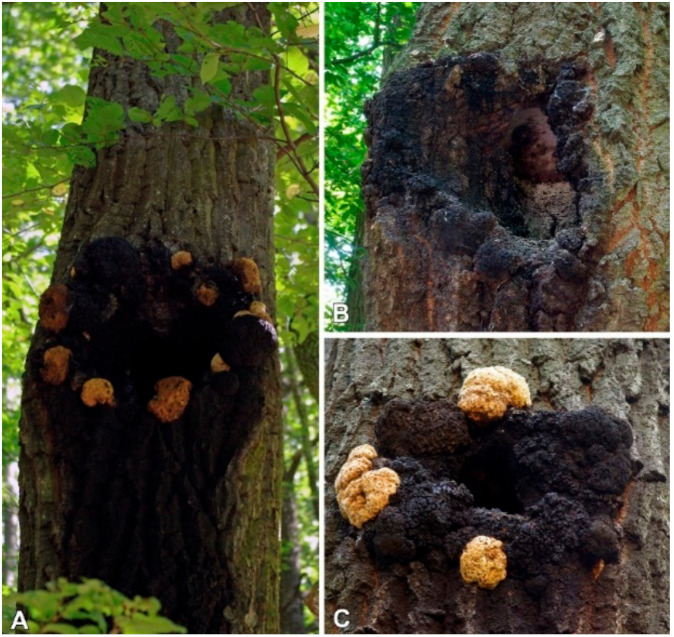
Basidocarp and the imperfect stage (sterile conk) of *Inonotus nidus-pici* on living *Quercus cerris* in Hungary. (**A**) Young and old sterile conks of the studied specimen (PV1043); (**B**) old sterile conks and sporulating basidiocarp (Börzsöny Mts, 19. Jun 2021; (**C**) young and old sterile conks (Visegrád Mts, 1 October 2011). Photos: V. Papp.

The so-called chaga mushroom (*Inonotus obliquus*) is a wood-decay fungus that produces a massive, black, crusty conk (i.e., sclerotium) mainly on living birch (*Betula* spp.) trees. The sterile conk of *Inonotus obliquus* (Ach. ex Pers.) Pilát is a well-known source of traditional medicine, especially in Russia, Poland, and the Baltic countries, and has a long history of use by indigenous people to treat cancer, cardiovascular diseases, viral and bacterial infections, and gastro-intestinal disorders [11,12,13]. Currently, *I. obliquus* is one of the most intensely studied medicinal mushrooms, and has been shown to contain a variety of biologically active compounds, e.g., polysaccharides, triterpenoids, and styrylpyrone derivatives [14,15,16,17]. In contrast, no data are available in the literature concerning the chemical constituents of a closely related species, *I. nidus-pici* Pilát ex Pilát. Despite this, in previous research we highlighted the antioxidant and xanthine oxidase activities of this fungus [18]. Based on the similar sterile conks produced by this species, *I. nidus*-*pici* was previously considered as only a form of *I*. *obliquus* and discussed in the former literature under the name *Poria obliqua* f. “sur chêne” [19], or *Xanthochrous obliquus* f. *cavernatus* [20]. However, the two species are well-separated by morphological features and phylogenetic evidence [21,22]. In contrast with *I. obliquus*, both the poroid form and the sterile conks of *I. nidus-pici* develop on the same host and colonize mainly living *Quercus* spp. (i.e., *Q. cerris*), or occasionally other angiosperms, e.g., *Fagus*, *Juglans*, *Aesculus*, *Platans*, and *Fraxinus* species [23,24]. The resupinate basidiomes of *I. nidus-pici* develop inside the cavities of the host tree, while the yellowish-brown sterile conks (becoming black, hard, and rimose) grow on cortical layers around the cavity (Figure 1). 

According to the literature, *I. nidus-pici* (*Hymenochaetaceae*, *Basidiomycota*) is considered to be a mainly Central-Eastern European species, but has also been identified in Spain and Iran [23,24,25]. Given the lack of any information on the chemical profile of *I. nidus-pici*, we decided to conduct a research project to explore the mycochemical and pharmacological potential of this species.

## 2. Results and Discussion

**Figure 2:**
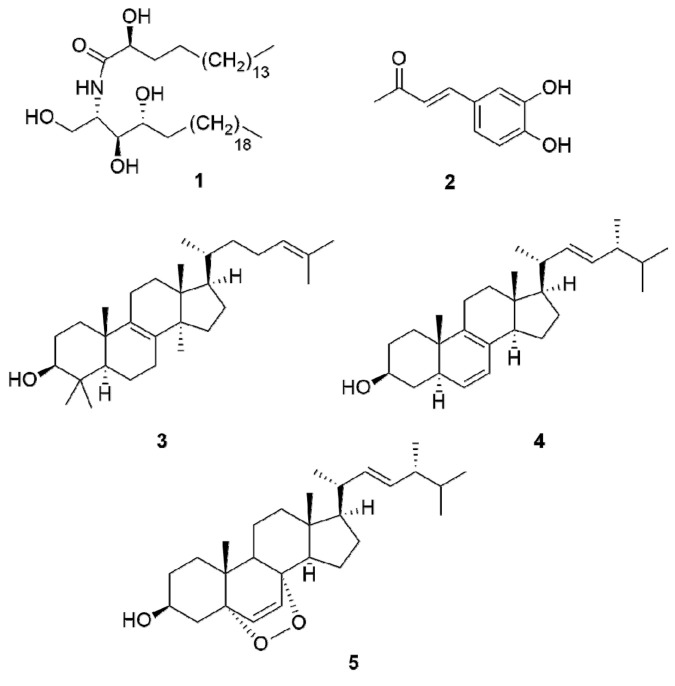
Compounds isolated from *Inonotus nidus-pici*.

Thorough investigation of the methanol extract obtained from the sterile conks of *I. nidus-pici* resulted in the identification of five compounds (Figure 2).

The fungal extract was first subjected to solvent–solvent partition between aqueous MeOH and *n*-hexane, followed by extraction with chloroform and ethyl acetate. The resulting *n*-hexane and chloroform extracts were purified using normal and reversed phase flash column chromatography. Compounds **1**–**5** were structurally characterized on the basis of NMR and MS spectroscopic data and confirmed by comparing them to those reported earlier in the literature.

All fungal metabolites isolated have been identified for the first time in sterile conks of *I. nidus-pici*. 

The ^1^H NMR spectrum of compound **1** in THF-*d*_8_ showed two overlapping resonances with triplet multiplicity at *δ* 0.89 (t, *J* = 7.0 Hz, 6H, CH_3_-18′, CH_3_-24) ppm which indicated the presence of two methyl groups (Table 1). The methylene resonances at *δ* 1.22–1.60 (m, ~68H, H-3′-H17′, H-5-H-23) indicated the presence of two saturated aliphatic chains. The ^1^H resonance at *δ* 7.45 (d, *J* = 8.5 Hz, ^1^H, NH-1′) ppm lacking ^13^C HSQC correlation and the ^13^C resonance at *δ* 175.1 ppm revealed the presence of an amide moiety. According to its correlation in the COSY spectrum, the amide group is adjacent to a CH group at *δ* 4.06 (m, 1H, H-2) ppm. The ^13^C NMR spectrum showed four well-separated resonances at *δ* 77.1 (C-3), 73.1 (C-4), 72.7 (C-2′), and 62.3 (C-1) ppm. These resonances and their HSQC correlations suggested the presence of a CH_2_ and three CH groups attached to hydroxyl groups. Moreover, the resonances of the four hydroxyl groups also appeared in the ^1^H NMR spectrum at *δ* 4.92 (d, *J* = 5.2 Hz, 1H, 2′-OH), 4.38 (m, 1H, 3-OH), 4.37 (m, 1H, 1-OH), and 3.98 (d, *J* = 6.4 Hz, 1H, 4-OH) ppm. Based on the aforementioned NMR characteristics combined with their homo- and heteronuclear correlations, the compound was suggested to be a ceramide derivative in which the fatty acid chain contains a hydroxyl group at the alpha position, and the long chain base contains three hydroxyl groups. Due to the overlapping methylene resonances, MS experiments were necessary to unequivocally confirm the exact structure. MS experiments showed that the accurate mass found for the [M + H]^+^ ion was 684.65127, while the calculated mass for [C_42_H_85_O_5_N + H]^+^ was 684.65005 in positive ESI mode resulted in a relative mass difference of 2.5 ppm. Mass accuracy was between 2.5 and 3.5 ppm for the fragment ions. The resulting protonated species [M + H]^+^ underwent a neutral loss (H_2_O), resulting in the fragment ion *m*/*z* 666 in the MS/MS spectrum. Further to this, a subsequent loss of water produced the fragment ion 648 (M + H − H_2_O − H_2_O = 648). A minor fragment ion peak (*m*/*z* 300; C_18_H_38_O_2_N) can prove the number of methylene groups next to the amide group (i.e., the number of carbon atoms in the fatty acid residue), while *m*/*z* 282 ion (C_18_H_36_ON) can be explained as water loss from *m*/*z* 300. The measured NMR and MS spectra for compound **1** can be found in the Supplementary Material (Table S1, Figures S1–S10).

Thus, the structure of compound **1** was identified as citropremide, which has a polyhydroxylated ceramide structure. It has been isolated for the first time from a fungal species, previously being identified only in the stem bark of the evergreen shrub *Citropsis gabonensis* from the *Rutaceae* family [26]. NMR spectra were also measured in pyridine-*d*_5_ for comparison with data in the previous literature (Figures S7 and S8). The identity of the NMR chemical shifts and the optical rotation of compound **1** ($\alpha_{D}^{26}$ + 8, pyridine, *c* 0.25) also suggested the same absolute configuration as the known compound: (2*S*,3*S*,4*R*)-2-[(2*R*)-2hydroxyoctadecanoylamino]tetracosane-1,3,4-triol. This configuration is the most typical for related ceramides from natural sources in which the fatty acid and the long chain base lengths vary, but the molecular formula is the same [27,28]. 

Compound **2** is 3,4-dihydroxybenzalacetone (osmundacetone by its vernacular name), and was previously identified in several medicinal mushrooms, e.g., *Inonotus* and *Phellinus* species [29,30], and also in the Far Eastern fern, *Osmunda japonica*, from where its name originates [31]. The NMR characteristics of compound **2** are provided in the Supplementary Material (Table S2, Figures S13 and S14), which are similar to the characteristics exhibited in previous publications [24].

Osmundacetone exhibits significant antioxidant properties. Moreover, it is neuroprotective based on its activity against oxidative glutamate toxicity in an HT22-immortalized hippocampal cell line, according to a recent study published by Trinh et al. [32].

Compound **3** was found to be a lanostane, while **4** and **5** were ergostane type triterpenes. The 1D and 2D NMR spectra of the isolated compounds suggested that they were lanosterol (**3**), ergost-6,8,22-trien-3β-ol (**4**), and ergosterol peroxide (**5**). The ^1^H NMR data of compound **3** was similar to previously published data, and the NMR spectra of **4** and **5** were also identical to that of a previous report [28,29]. The complete ^1^H and ^13^C resonance assignments of the compounds are given in the Supplementary Material (Table S3, Figures S15–S20).

In the used concentrations, compounds **1** and **5** were inactive in the antimicrobial and antioxidant assays. An antioxidant effect was observed only in the case of **2**, with EC50 29.7 ± 1.3 µM that was similar to the (+)-catechin positive controls EC50 14.6 ± 0.4 µM. Neither compound **2** nor **4** could inhibit mycelial growth of the tested fungi at the used concentration, but they showed moderate antibacterial activity (Table 2). Osmundacetone (**2**) was effective against all bacterial strains except *B. subtilis*, while compound **4** inhibited all bacterial strains except *A. fischeri*. Comparing their antibacterial potencies to those of antibiotics, the strongest activity was attributed to compound **4** against *B. subtilis* with a five times higher MIC value than that of gentamicin (Table 2).

The cytotoxicity experiments revealed that among the isolated compounds, ergosterol peroxide (**5**) was the most effective metabolite, providing the lowest IC50 (36.18 µM) value against the doxorubicin resistant cell line, being comparable with the IC50 of 18.4 µM of the reference compound, doxorubicin (Table 3). Compared to the triterpenes (**4**–**5**) and the phenolic compound (**2**), citropremide (**1**) exhibited a noticeably decreased activity against all the investigated human cancer cell lines. Considering the results of compound **4**, a substantially decreased cytotoxic property was detected against the epidermoid cancer cell line. In general, among the three examined cell lines, MES-SA proved to be the most susceptible to the applied fungal metabolites.

## 3. Materials and Methods

Optical rotation was measured on a Jasco P-2000 digital polarimeter at the Na_D_ line. The chemicals used in the experiments were supplied by Sigma-Aldrich, Budapest, Hungary and Molar Chemicals, Halásztelek, Hungary. Flash chromatography was carried out on a CombiFlash^®^ Nextgen 300+ instrument with 200–800 nm UV-VIS variable wavelength detection using RediSep Rf Gold normal-phase silica and reversed-phase C18 Flash columns (4, 12, 40 and 60 g) (Teledyne Isco, Lincoln, NE, USA). NMR spectra were recorded in deuterated chloroform (chloroform-*d*, 99.8 atom% D, contains 0.03% (*v*/*v*) TMS, Sigma-Aldrich), methanol (methanol-*d*_4_, 99.8 atom% D, contains 0.03% (*v*/*v*) TMS, Sigma-Aldrich), tetrahydrofuran (tetrahydrofuran-*d*_8_, 99.5 atom% D, Sigma-Aldrich) or pyridine (pyridine-*d*_5_, 99.8 atom% D, Sigma-Aldrich) on a BRUKER AVANCE III HD 600 (600/150 MHz) instrument equipped with a Prodigy cryo-probehead at 295 K. The pulse programs were taken from the Bruker software library (TopSpin 3.5). ^13^C and ^1^H chemical shifts (*δ*) are given in ppm relative to the NMR solvent or relative to the internal standard (tetramethylsilane), while the coupling constants (*J*) are given in Hz. The complete ^1^H and ^13^C assignments were achieved by widely accepted strategies based on ^1^H NMR, ^13^C NMR, ^1^H-^1^H COSY, ^1^H-^1^H NOESY, ^1^H-^13^C HSQC, and ^1^H-^13^C HMBC measurements. HRMS analyses were performed on a Thermo Velos Pro Orbitrap Elite (Thermo Fisher Scientific, Bremen, Germany) system. The ionization method was EI (with 70 eV) and ESI operated in positive ion mode. The protonated molecular ion peaks were fragmented by CID at a normalized collision energy of 35–45%. For the CID experiment, helium was used as the collision gas. The samples were dissolved in methanol. Data acquisition and analysis were accomplished with Xcalibur software version 4.0 (Thermo Fisher Scientific, Bremen, Germany).

### 3.1. Mushroom Material

Sterile conks of *Inonotus nidus-pici* were collected in 2014 in Börzsöny Mts, near Király-rét, on living *Quercus cerris*; 47.906553, 18.999720, Hungary. Fungal identification was made by Viktor Papp. A voucher specimen (No. PV 1043) has been deposited at the Department of Botany, Hungarian University of Agriculture and Life Sciences, Hungary. The fungal sample collected was carefully cleaned, all contaminating materials, i.e., any plant part (e.g., bark) and/or soil contaminant were removed before processing. The samples were dried at room temperature for five days and stored in a cool and dry place until extraction.

### 3.2. Extraction and Isolation

The air-dried sterile conks (302 g) of *I. nidus-pici* were ground, and then extracted with 5 × 1500 mL methanol for 5 × 90 min using an ultrasonic bath. Following filtration, the fungal extracts were combined and concentrated in a vacuum. The dry residue (10.1 g) was dissolved in 300 mL of 50% aqueous MeOH and was subjected to liquid–liquid partition between *n*-hexane (4 × 150 mL), CHCl_3_ (4 × 150 mL) and ethyl acetate (4 × 150 mL). The *n*-hexane fraction (2.12 g) was subjected to flash chromatography on a silica gel column using a gradient system of *n*-hexane and acetone (0–25%; t = 50 min). Fractions with similar compositions were combined according to TLC monitoring (A1–A18). From the combined fraction A15 (29 mg), a precipitation formed, which was washed with *n*-hexane and then acetone to obtain compound **1** (19 mg). Combined fraction A4 (208 mg) was further purified in consecutive steps of normal (gradient system of hexane–acetone) and reversed phase (eluent system of water–methanol) flash chromatography steps to give compounds **3** (3 mg) and **5** (5 mg). Another fraction (A7, 142 mg) was subjected to a final purification by normal- (eluent system of *n*-hexane–acetone) and reversed- (eluent system of water–methanol) phase flash chromatography, leading to compound **4** (14 mg). The chloroform soluble phase (1.14 g) was subjected to flash chromatography on silica gel column using gradient system of *n*-hexane–acetone (5–45%; t = 55 min). Fractions with similar compositions were combined according to TLC monitoring (B1–B11). Fraction B9 (71 mg) was further separated by combination of normal- (*n*-hexane–acetone) and reversed- (water–methanol) phase flash chromatography steps to obtain compound **2** (18 mg).

### 3.3. Antimicrobial Assays

Isolated compounds **2** (4 mg/mL), **4** (2 mg/mL), and **5** (2 mg/mL) were dissolved in ethanol. Gram-positive *Bacillus subtilis* subsp. *spizizenii* soil bacterium (DSM 618) was from Merck (Kenilworth, New Jersey), and *Rhodococcus fascians* (B.01608) was purchased from the National Collection of Agricultural and Industrial Microorganisms (NCAIM, Budapest, Hungary). Gram-negative, naturally luminescent marine bacterium *Aliivibrio fischeri* (DSM 7151) were obtained from the Leibniz Institute DSMZ–German Collection of Microorganisms and Cell Cultures, Berlin, Germany. Luminescent *Arabidopsis* pathogen *Pseudomonas syringae* pv. *maculicola*, chromosomally tagged with lux-CDABE genes, was kindly provided by Jun Fan (John Innes Center, Department of Disease and Stress Biology, Norwich, UK). *F. avenaceum* strain IMI 319,947 was from CABI-IMI Culture Collection, Egham, UK, and *Bipolaris sorokiniana* (Sacc.) Shoemaker H-299 (NCBI GenBank accession No. MH697869) was collected in Hungary. *B. subtilis* in Lysogeny broth (10 g/L tryptone (Reanal, Budapest, Hungary), 5 g/L yeast extract (Scharlau, Barcelona, Spain) and 10 g/L sodium chloride (Reanal)) at 37 °C, *R. fascians* in Waksman broth (5 g/L peptone (Scharlau), 5 g/L meat extract (Reanal), 5 g/L sodium chloride (Reanal), 10 g/L glucose (Reanal) at 26 °C, concentrated sodium hydroxide (Reanal) solution to set the pH at around 7.4), *A. fischeri* in ocean medium (5 g/L tryptone, 3 g/L yeast extract, 3 g/L glycerol (Reanal), 35 g/L sea salt mix (Instant Ocean), concentrated sodium hydroxide (Reanal) solution to set the pH at around 7.2) at 28 °C, and *P. maculicola* in gelatin broth (20 g/L pancreatic digested gelatin (Carl Roth, Karlsruhe, Germany), 10 g/L potassium sulfate (Reanal), 10 mL/L glycerol (Reanal), 1.4 g/L magnesium chloride (Reanal)) at 28 °C were grown by shaking at 120 rpm. *F. avenaceum* and *B. sorokiniana* were grown in Lysogeny broth by shaking at 120 rpm at 21 °C for 3 days in the dark. The washed mycelium was cut in LB to small pieces with a FastPrep^®^-24 Classic homogenizer (7 × 2 mm glass beads in a 2-mL Eppendorf tube, 4.5 m/s for 20 s, MP Bio, Beograd, Serbia).

96-well microplates were used for the determination of the minimal inhibitory concentration (MIC) and the half maximal inhibitory concentration (IC_50_) of the isolates against the microbial growth. Gentamicin and chloramphenicol (both from Sigma) were used as the positive control, and ethanol was used as the negative control. Two-fold dilution series of 5 µL of the isolates (made in triplicate) in ethanol was prepared in the microtiter plates. After the evaporation of the ethanol in a sterile box, 70 µL of LB and 50 µL of a mycelium suspension (OD600 0.2) or 150 µL of bacterial suspension (10^5^ CFU/mL) were added to each well. The absorbance at 600 nm was measured by a microplate spectrophotometer (Labsystems Multiscan MS 4.0, Thermo Scientific, Waltham, MA, USA) immediately before and after incubation (24 h for bacteria by shaking with Grant PHMP microplate shaker and 72 h for fungi). The experiment was repeated on two separate occasions with three parallels, and the results were averaged. Minimal bactericide concentration (MBC) was determined by plotting 5 µL of each well onto appropriate agar layers, and after incubation the presence or absence of bacterial colonies was checked.

### 3.4. DPPH Assay

Antioxidant assay was performed in 96-well microplates using 2,2-diphenyl-1-picrylhydrazyl radical (DPPH**˙**, Sigma, St. Louis, MO, USA) and two-fold dilution of the samples. The positive control was (+)-catechin (Sigma) dissolved in ethanol (1 mg/mL). In the wells, the samples were mixed with 75 µL ethanol and 50 µL ethanolic DPPH**˙** solution (0.2 mg/mL). The absorbance at 492 nm was measured by a microplate reader (Labsystems Multiscan MS 4.0) after 30 min incubation at room temperature in the dark. The experiment was repeated three times, EC50 (half maximal effective concentration) was determined, and the results were averaged.

### 3.5. Cytotoxic Assay

The MES-SA (No.: CRL-1976™) uterine sarcoma cell line and its doxorubicin-selected derivative MES-SA/Dx5 (No.: CRL-1977™) were purchased from ATCC. A431 human skin-derived, epidermoid carcinoma cell line was purchased from ATCC.

After incubation with the compounds (diluted from 10 mM stock solutions in DMSO, except for Compound **1**, which was dissolved in THF), which lasted for 72 h (the initial seeded cell number was at a density of 5000 cells/well), the supernatant was removed, washed once with DPBS, and the viability was assessed by means of the PrestoBlue^®^ assay (Invitrogen™, USA; purchased from Thermo Fisher Scientific, Waltham, MA, USA), according to the manufacturer’s instructions. Viability of the cells was measured spectrophotometrically (measuring fluorescence, excitation at 560 nm and emission at 590 nm) using an EnSpire microplate reader (Perkin Elmer, Waltham, MA, USA). Curve fit statistics were used to determine the concentration of the test compound that resulted in 50% toxicity (IC_50_). Curves were fitted by using the sigmoidal function (Origin) or sigmoidal dose–response (comparing variable and fixed slopes) model (GrapPad Prism), and they were expressed as the mean of three independent experiments (each one performed with three replicates). Data were normalized to untreated cells. The IC_50_ value (given in µM) is the average of at least three independent determinations. Every IC_50_ value represents the corresponding GI_50_ value (the average growth inhibition of 50%).

## 4. Conclusions

The current study represents the first mycochemical examination of the poroid fungus *I. nidus-pici*, a disregarded relative of the well-known medicinal mushroom *Inonotus obliquus*. In-depth chemical analysis of the methanol extract of *I. nidus-pici* led to the identification of a ceramide (1), a phenolic derivative (2), and three triterpene steroids (3–5). The pharmacological experiments that we conducted revealed that compounds **2** and **4** exhibited moderate antimicrobial properties, that 2 showed notable antioxidant property in DPPH assay, while compounds **4** and **5** exerted significant antiproliferative properties on three human cancer cell lines, including a doxorubicin resistant one.

## Figures and Tables

**Table 1:** Complete ^1^H and ^13^C NMR resonance assignments for compound **1** (THF-*d*_8_).

	No.	Compound 1		
long chain base		*δ* ^13^C	*δ* ^1^H	multiplicity
	1	62.3	3.74	dt, *J* = 10.8, 4.4 Hz
			3.65	m
	1-OH	-	4.37	m
	2	53.1	4.06	m
	3	77.1	3.42	m
	3-OH	-	4.38	m
	4	73.1	3.44	m
	4-OH	-	3.98	d, *J* = 6.4 Hz
	5	34.3	1.71	m
			1.33	m
	6–23	23.6–32.9	1.22–1.60	m
	24	14.5	0.89	t, *J* = 7.0 Hz
fatty acid	NH-1′	-	7.45	d, *J* = 8.5 Hz
	1′	175.1	-	-
	2′	72.7	3.92	m
	2′-OH	-	4.92	d, *J* = 5.2 Hz
	3′	35.9	1.74, 1.53	m
	4′	26.2	1.42	m
	5′–17′	23.6–32.9	1.22–1.60	m
	18′	14.5	0.89	m

**Table 2:** The MBC, MIC and IC_50_ values of compounds **2** and **4** and two antibiotics in µM against 4 bacterial strains.

Strain	Compound 2			Compound 4			Gentamicin			Chloramphenicol	
	MBC	MIC	IC_50_	MBC	MIC	IC_50_	MBC	MIC	IC_50_	MBC	MIC
1	>1500	>1500	>1500	42.2	42.2	35.1 ± 0.2	6.9	6.9	0.71 ± 0.06	n.m.	n.m.
2	748.9	748.9	101.1 ± 2.8	168.4	168.4	41.9 ± 0.5	6.9	3.5	2.60 ± 0.15	n.m.	n.m.
3	>1500	>1500	882.0 ± 56.2	>336.7	>336.7	203.0 ± 8.1	8.8	8.8	1.09 ± 0.08	n.m.	n.m.
4	93.8	93.8	68.5 ± 2.8	>336.7	>336.7	>336.7	n.m.	n.m.	n.m.	1.24	1.24

^1^ Strains: (**1**) Bacillus subtilis subsp. spizizenii (Gram +), (**2**) Rhodococcus fascians (Gram +), (**3**) Pseudomonas syringae pv. maculicola (Gram −), (**4**) Aliivibrio fischeri (Gram −). n.m.—not measured.

**Table 3:** IC_50_ cytotoxicity values (µM) of isolated compounds and the reference compound against uterine sarcoma (MES-SA, MES-SA/Dx5) and epidermoid carcinoma (A431) cell lines.

Compound	MES-SA	MES-SA/Dx5	A431
**1**	92.1 ± 1.1	195.0 ± 9.8	142.1 ± 3.2
**2**	57.5 ± 3.1	109.6 ± 5.2	101.3 ± 4.1
**4**	41.9 ± 2.9	42.2 ± 2.1	83.2 ± 5.4
**5**	37.6 ± 2.6	36.2 ± 3.2	45.7 ± 3.9
*doxorubicin*	0.65 ± 0.04	18.4 ± 1.98	1.12 ± 0.11

## Data Availability

All data are found in the Supplementary Materials.

## Supplementary Materials

The following are available online: Figures S1–S10: NMR and MS spectra and spectral data of compound **1**, Figures S11–S18: NMR spectra and spectral data of compounds **2**–**5**, Table S1: HRMS spectral data of compound **1**, Table S2: Complete ^1^H and ^13^C NMR resonance assignments for compound **2**, Table S3: Complete ^1^H and ^13^C NMR resonance assignments for compounds **3**, **4** and **5**.

## Sample Availability

Samples of the Compounds **1**–**2**, **4**–**5** are available from the authors.
