# Supplementary material for: Antimicrobial, Antioxidant and Antiproliferative Secondary Metabolites from Inonotus nidus-pici
Source: Molecules. 2021 Sep 7;26(18):5453. doi: 10.3390/molecules26185453 (PMC8468361; doi:10.3390/molecules26185453)
Supplement: Supplementary file 1 [file molecules-26-05453-s001.zip › molecules-1347549-supplementary.pdf]

Antimicrobial, antioxidant and antiproliferative secondary metabolites from *Inonotus nidus-pici*

Zsófia Garádi[1,2], Miklós Dékány [3], Ágnes M. Móricz[4], Anikó Gaál[5], Viktor Papp[6], Szabolcs Béni[1], and Attila Ványolós[1,*]

[1] *Department of Pharmacognosy, Semmelweis University, Üllői u. 26, H-1085 Budapest, Hungary; garadi.zsofia@pharma.semmelweis-univ.hu, beni.szabolcs@pharma.semmelweis-univ.hu,*
[2] *Directorate of Drug Substance Development, Egis Pharmaceuticals Plc, P. O. Box 100, H-1475 Budapest, Hungary;*
[3] *Spectroscopic Research, Gedeon Richter Plc., Gyömrői út 19-21, H-1103 Budapest, Hungary; m.dekany@richter.hu*
[4] *Plant Protection Institute, Centre for Agricultural Research, ELKH, 1022 Budapest, Herman Ottó út 15, Hungary; moricz.agnes@atk.hu*
[5] *Biological Nanochemistry Research Group, Institute of Materials and Environmental Chemistry, Research Centre for Natural Sciences, Magyar Tudósok Körútja 2, H-1117 Budapest, Hungary, gaal.aniko@ttk.hu*
[6] *Department of Botany, Hungarian University of Agriculture and Life Sciences, H-1118 Budapest, Villányi út 29-43, Hungary; papp.viktor@uni-mate.hu*

*Correspondence: vanyolosa@pharmacognosy.hu

Table of contents

NMR and MS spectra and spectral data of compound **1** ... 3

Figure S1. ^{1}H NMR spectrum of compound **1** (THF-d_8) ... 3

Figure S2. ^{13}C spectrum of compound **1** (THF-d_8) ... 3

Figure S3. COSY spectrum of compound **1** (THF-d_8) ... 4

Figure S4. HSQC spectrum of compound **1** (THF-d_8) ... 4

Figure S5. HMBC spectrum of compound **1** (THF-d_8) ... 5

Figure S7. ^{1}H NMR spectrum of compound **1** (pyridine-d_5) ... 6

Figure S8. ^{13}C NMR spectrum of compound **1** (pyridine-d_5) ... 6

Figure S9. HRMS spectrum of compound **1** ... 7

Figure S10. Full HRMS spectrum of compound **1** ... 7

Figure S11. HR-MS/MS spectrum of compound **1** ... 8

Table S1. HRMS spectral data of compound **1** ... 8

Figure S12. Mass fragmentation of compound **1** ... 8

NMR spectra and spectral data of compound **2** ... 9

Table S2. Complete ^{1}H and ^{13}C NMR resosnance assignments for compound **2** (methanol-d_4) ... 9

Figure S13.^{1}H NMR spectrum of compound **2** (methanol-d_4) ... 10

Figure S14.^{13}C NMR spectrum of compound **2** (methanol-d_4) ... 10

NMR spectra and spectral data of compound **3, 4 and 5** ... 11

Table S3. Complete ^{1}H and ^{13}C NMR resosnance assignments for compounds **3, 4 and 5** ($CDCl_3$) ... 12

Figure S15.^{1}H NMR spectrum of compound **3** ($CDCl_3$) ... 13

Figure S16.^{13}C NMR spectrum of compound **3** ($CDCl_3$) ... 13

Figure S17.^{1}H NMR spectrum of compound **4** ($CDCl_3$) ... 14

Figure S18.^{13}C NMR spectrum of compound **4** ($CDCl_3$) ... 14

Figure S19.^{1}H NMR spectrum of compound **5** ($CDCl_3$) ... 15

Figure S20.^{13}C NMR spectrum of compound **5** ($CDCl_3$) ... 15

NMR and MS spectra and spectral data of compound **1**

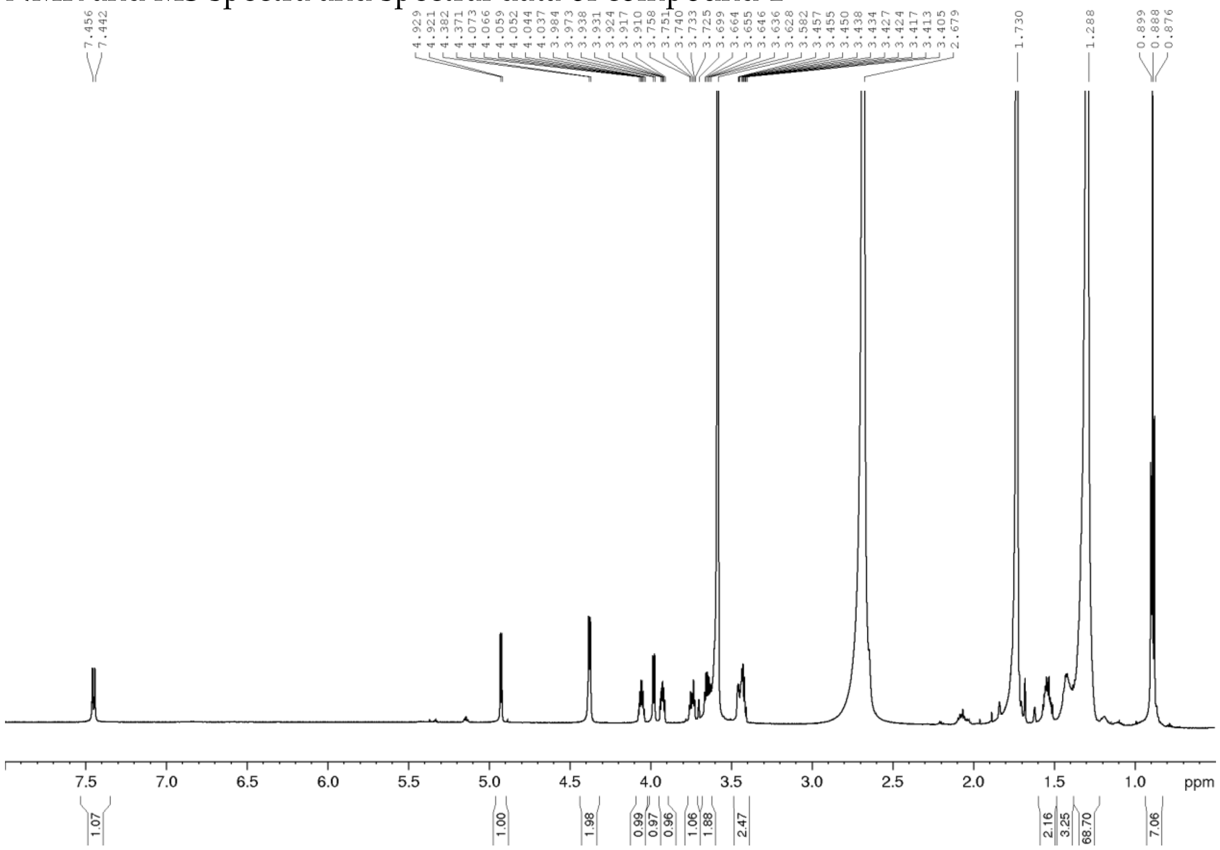

Figure S1. ^{1}H NMR spectrum of compound **1** (THF-d_8)

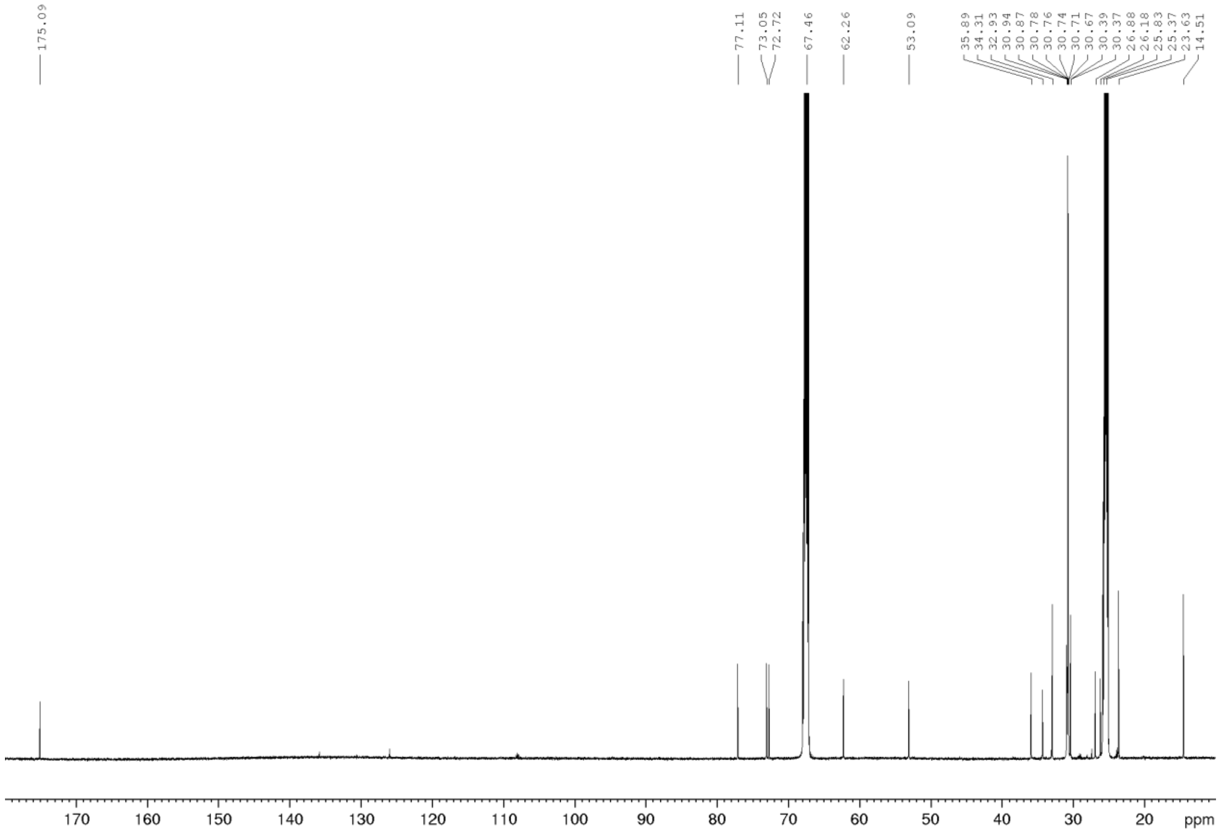

Figure S2. ^{13}C spectrum of compound **1** (THF-d_8)

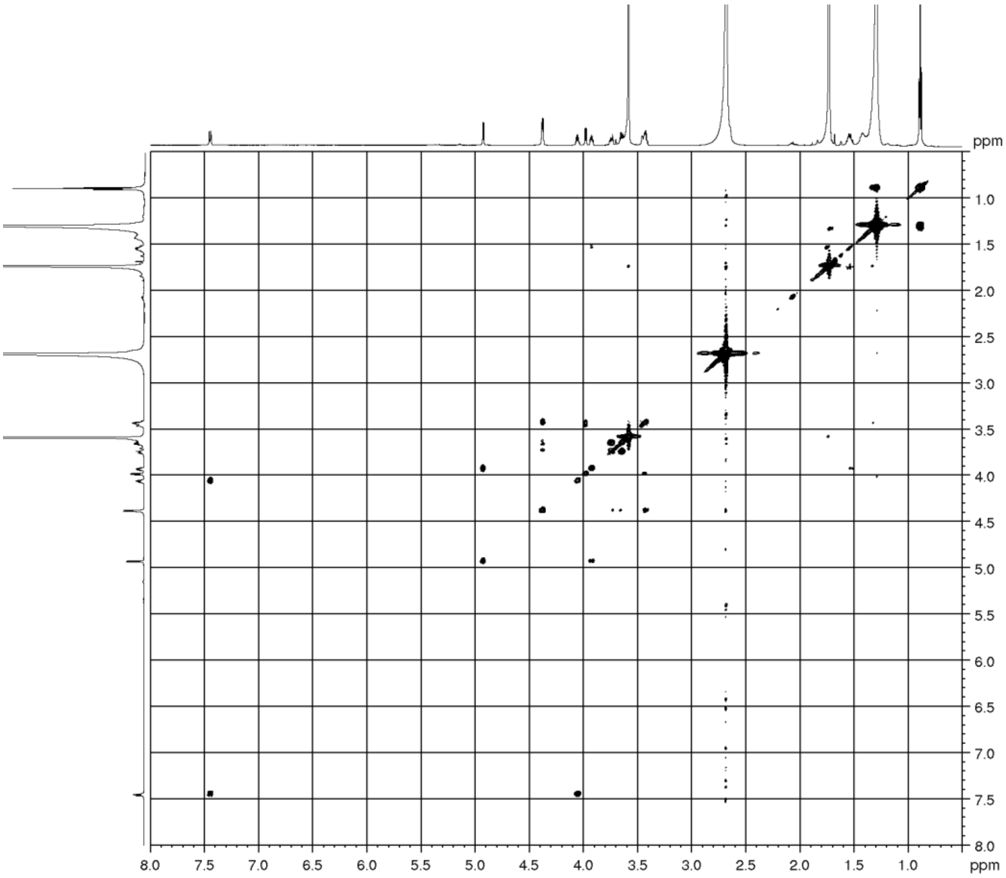

Figure S3. COSY spectrum of compound **1** (THF-d_8)

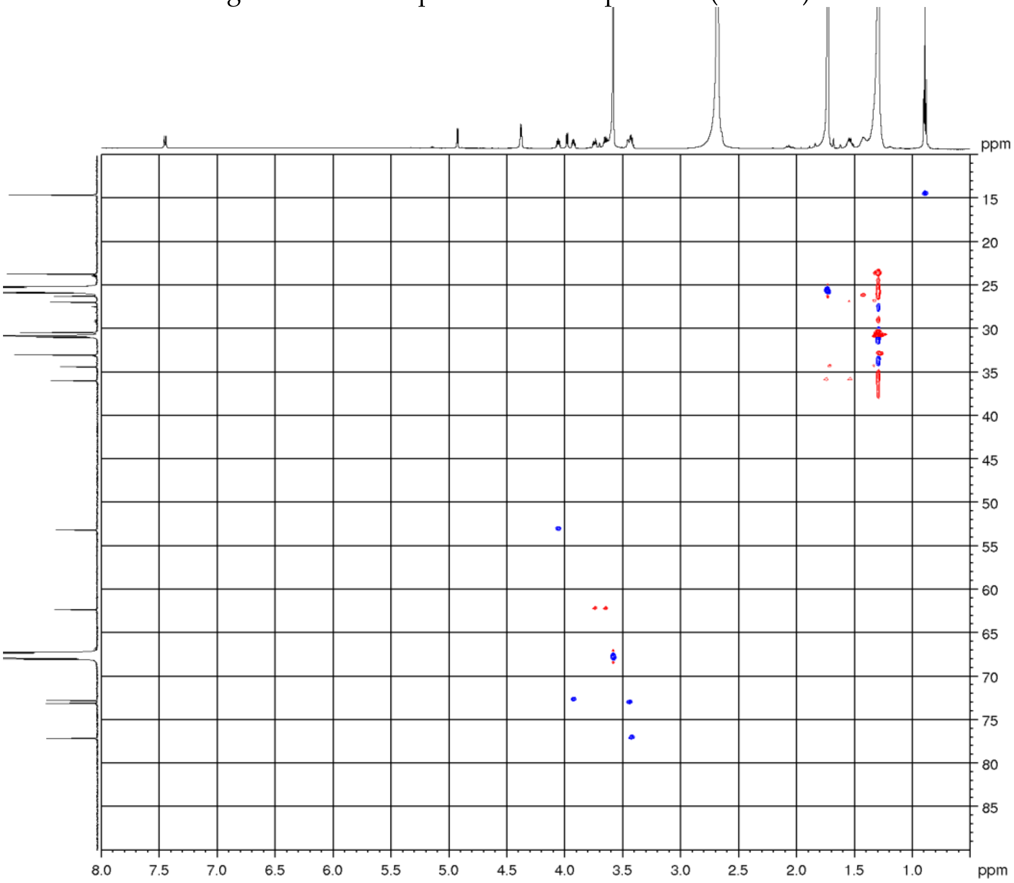

Figure S4. HSQC spectrum of compound **1** (THF-d_8)

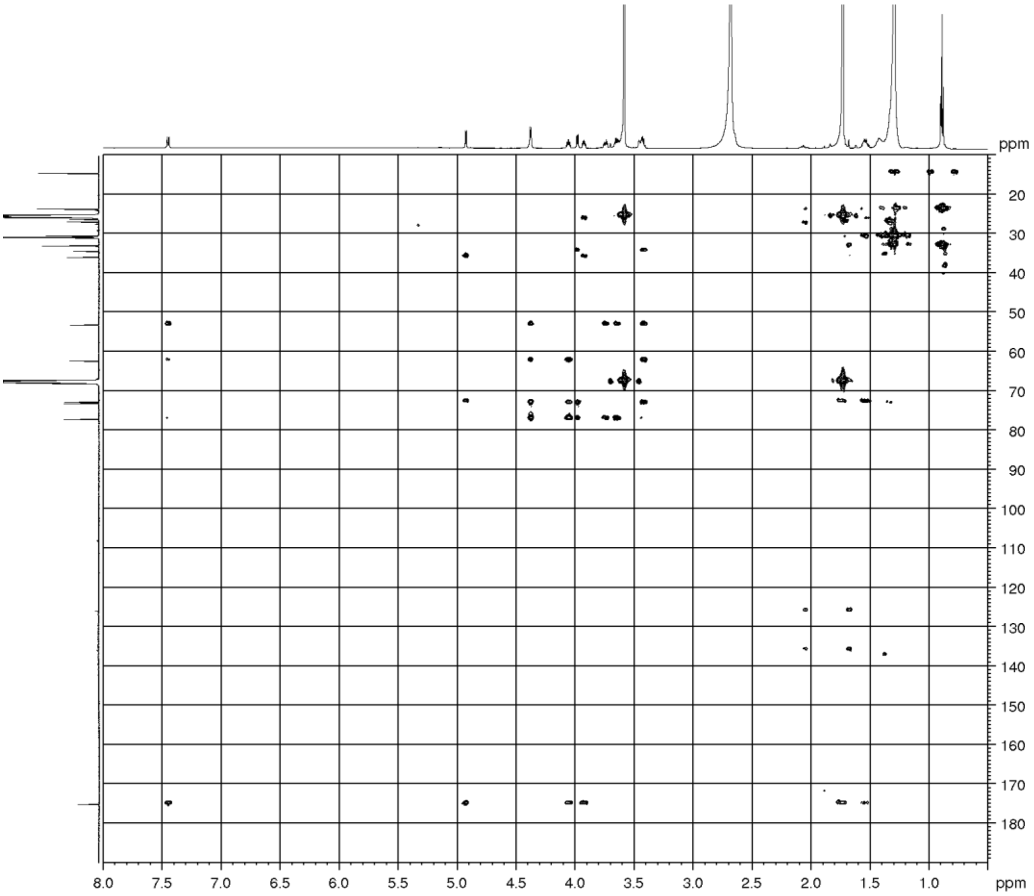

Figure S5. HMBC spectrum of compound **1** (THF-d_8)

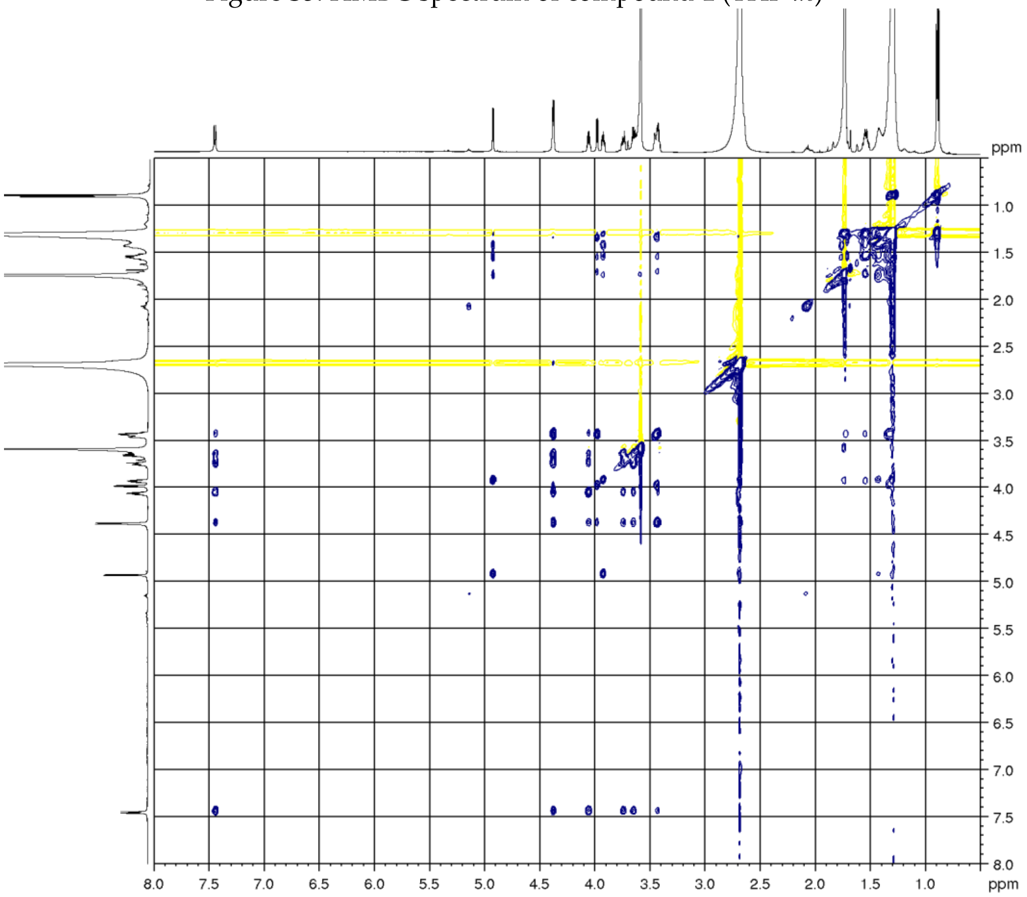

Figure S6. TOCSY spectrum of compound **1** (THF-d_8)

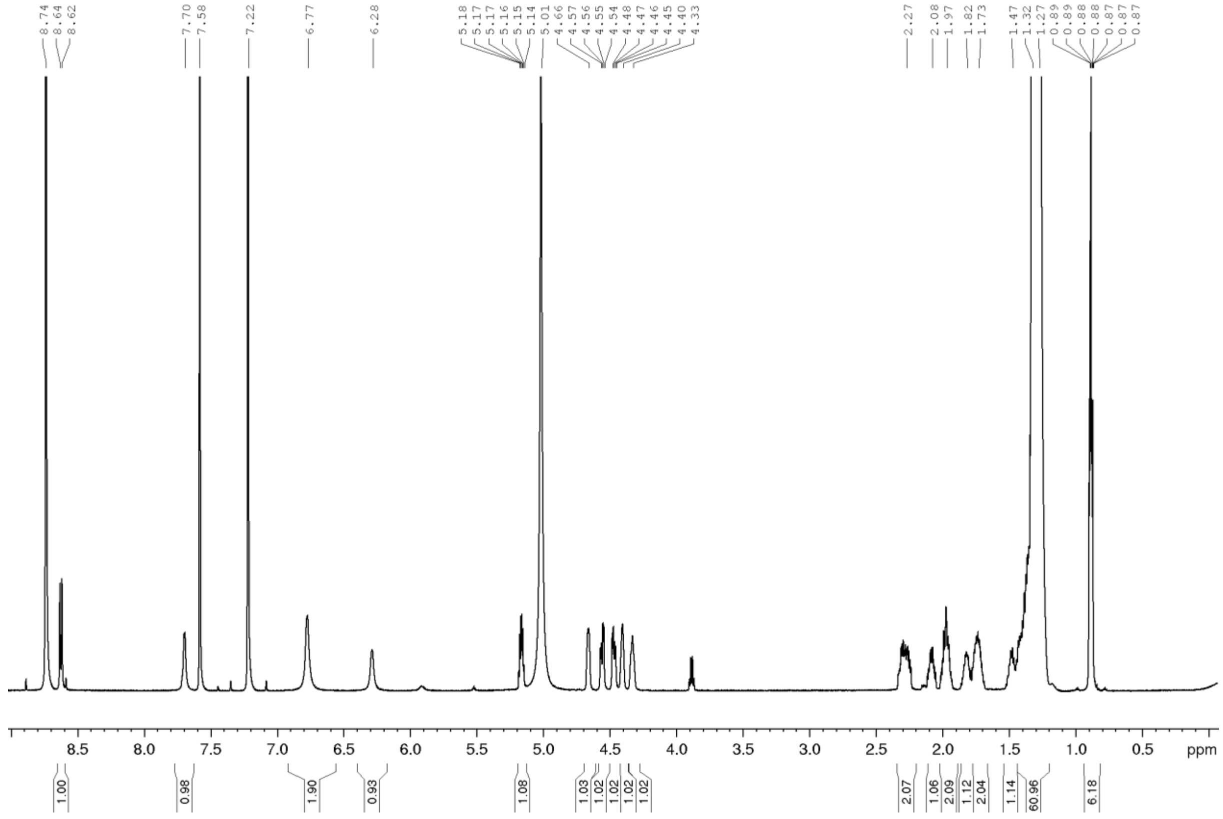

Figure S7. ^{1}H NMR spectrum of compound **1** (pyridine-d_5)

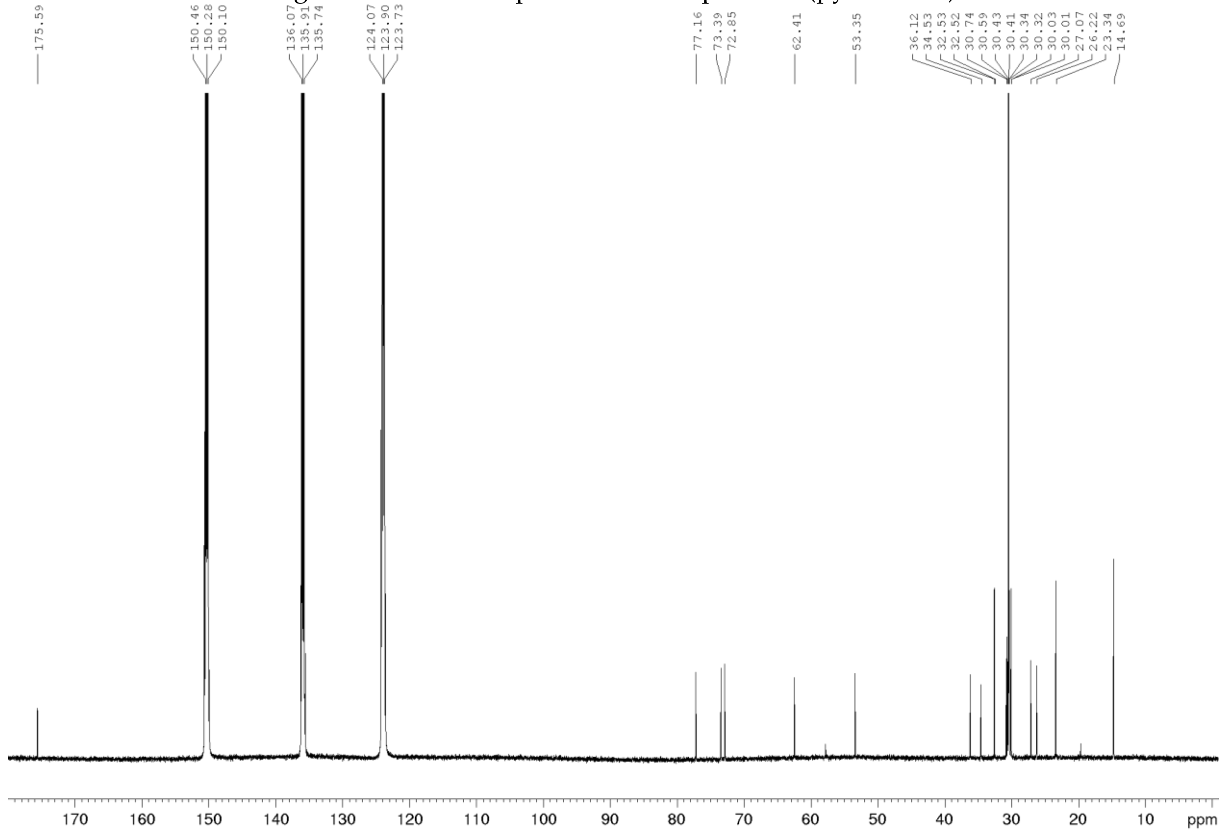

Figure S8. ^{13}C NMR spectrum of compound **1** (pyridine-d_5)

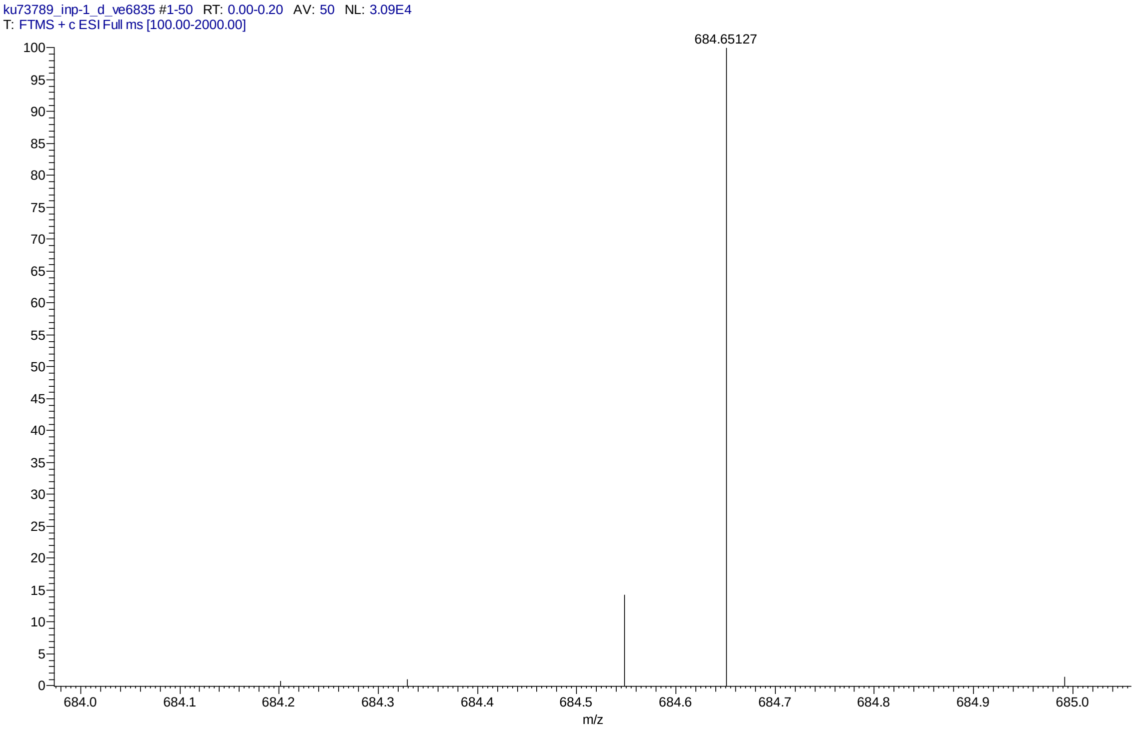

Figure S9. HRMS spectrum of compound **1**

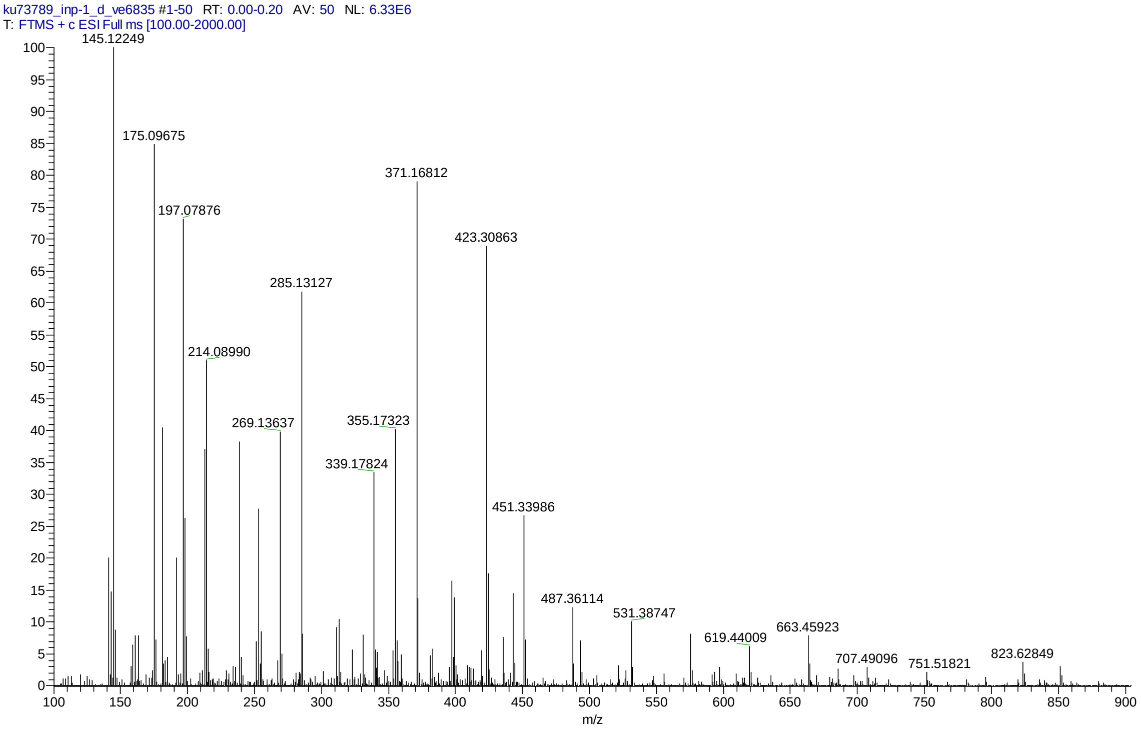

Figure S10. Full HRMS spectrum of compound **1**

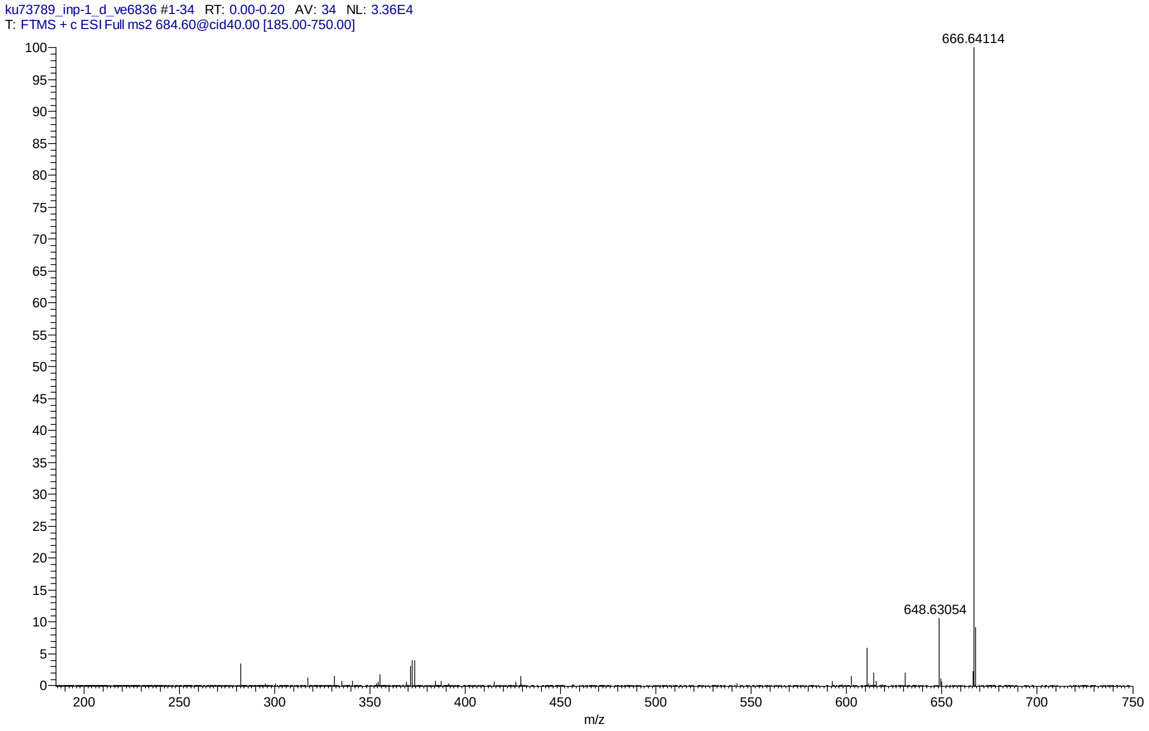

Figure S11. HR-MS/MS spectrum of compound **1**

HRMS: M+H=684.65127 (delta=1.8 ppm; $C_{42}H_{86}O_5N$). HR-ESI-MS-MS (CID=40%; rel. int. %): 666(100); 648(11); 300(<1); 282(3).

666.64114	666.63949	2.48	1.5	C42 H84 O4 N
648.63054	648.62892	2.49	2.5	C42 H82 O3 N
300.29071	300.28971	3.34	0.5	C18 H38 O2 N
282.28014	282.27914	3.54	1.5	C18 H36 O N

Table S1. HRMS spectral data of compound **1**

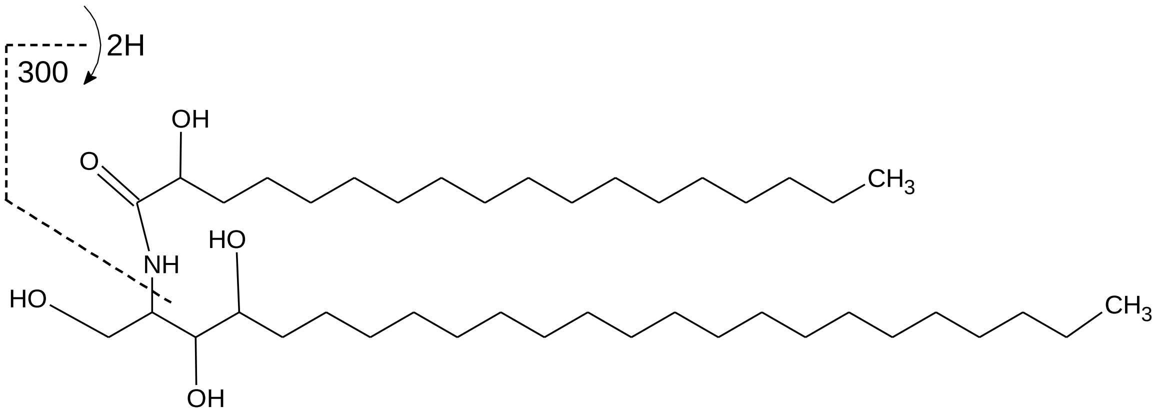

Figure S12. Mass fragmentation of compound **1**

NMR spectra and spectral data of compound **2**

No.	Compound 2		
	δ ^{13}C	δ ^{1}H	multiplicity
**1**	127.7	-	-
**2**	115.3	7.08	d (*J*=1.6 Hz)
**3**	146.9	-	-
**4**	150.0	-	-
**5**	116.5	6.80	d (*J*=8.2 Hz)
**6**	123.5	7.00	dd (*J*=8.2, 1.6 Hz)
**7**	146.9	7.52	d (*J*=16.2 Hz)
**8**	124.7	6.54	d (*J*=16.2 Hz)
**9**	201.6	-	-
**10**	27.0	2.33	s

Table S2. Complete ^{1}H and ^{13}C NMR resosnance assignments for compound **2** (methanol-d_4)

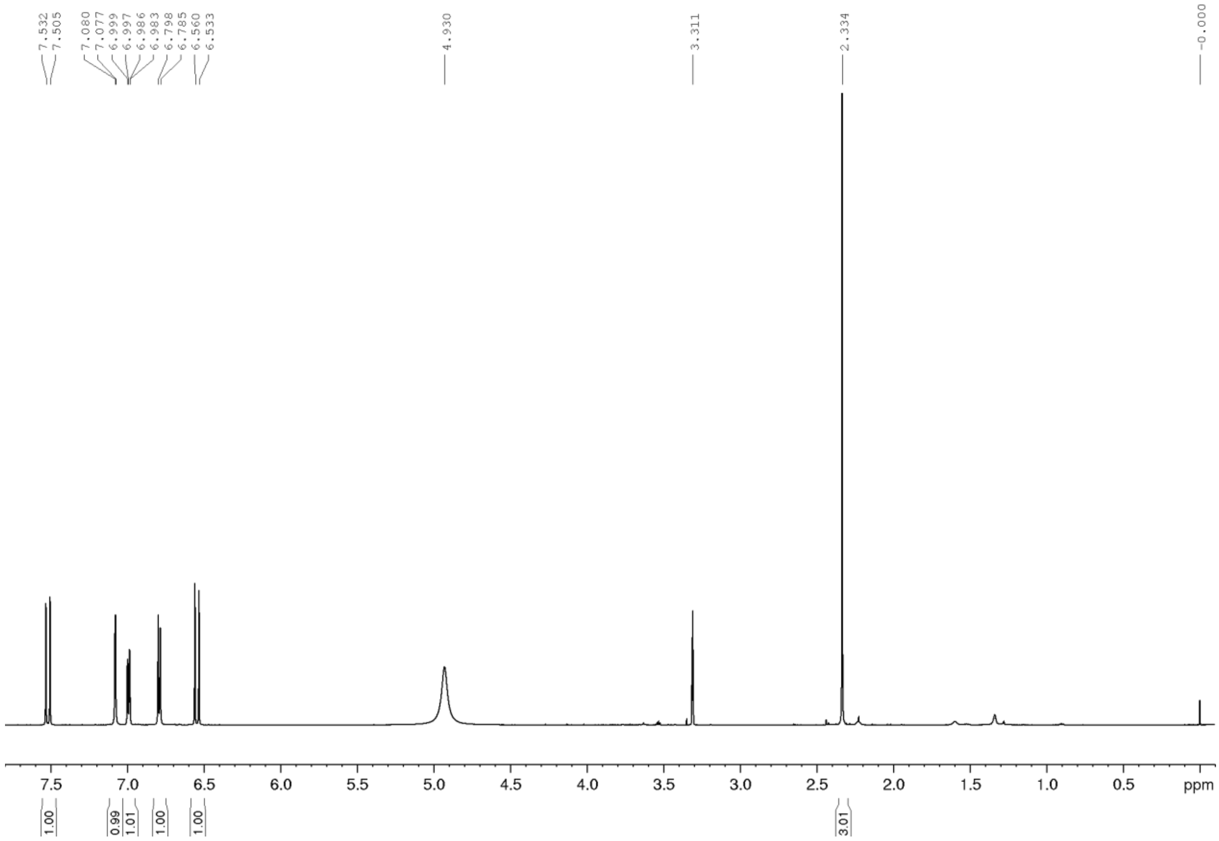

Figure S13.¹H NMR spectrum of compound **2** (methanol-d_4)

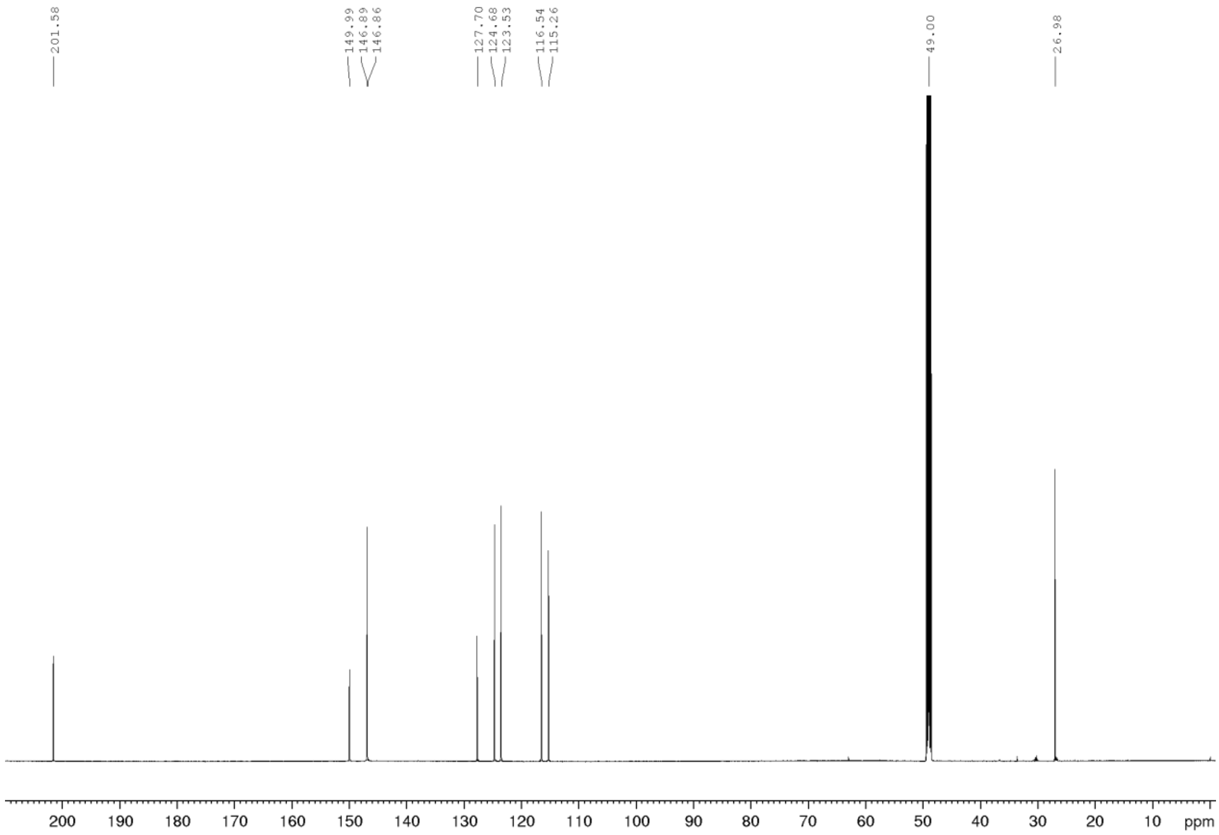

Figure S14.¹³C NMR spectrum of compound **2** (methanol-d_4)

NMR spectra and spectral data of compound **3, 4 and 5**

No.	Compound 3			Compound 4			Compound 5		
	**δ ^{13}C**	**δ ^{1}H**	**multiplicity**	**δ ^{13}C**	**δ ^{1}H**	**multiplicity**	**δ ^{13}C**	**δ ^{1}H**	**multiplicity**
**1**	35.6	1.73	m	38.3	1.89	m	34.6	1.95	m
		1.23	m		1.31	m		1.70	m
**2**	27.8	1.66	m	32.0	1.88	m	30.1	1.84	m
		1.59	m		1.50	m		1.54	m
**3**	79.0	3.24	dd, *J*=11.7, 4.4 Hz	70.4	3.64	m	66.5	3.97	m
**4**	38.9	-	-	40.8	2.47	m	36.9	2.11	m
					2.29	m		1.91	m
**5**	50.4	1.05	m	46.2	1.97	m	82.1	-	-
**6**	18.2	1.68	m	119.6	5.57	dd, *J*=5.8, 2.5 Hz	135.4	6.24	d, *J*=8.5 Hz
		1.51	m						
**7**	26.5	2.04	m	116.3	5.39	m	130.7	6.51	d, *J*=8.5 Hz
**8**	134.4	-	-	141.4	-	-	79.4	-	-
**9**	134.4	-	-	139.8	-	-	51.0	1.50	m
**10**	37.0	-	-	37.0	-	-	36.9	-	-
**11**	21.0	2.00	m	21.1	1.72	m	23.4	1.51	m
					1.59	m		1.23	m
**12**	30.9	1.69	m	39.0	2.06	m	39.3	1.95	m
					1.26	m		1.23	m
**13**	44.4	-	-	42.8	-	-	44.5	-	-
**14**	49.8	-	-	54.5	1.89	m	51.6	1.22	m
**15**	30.8	1.59	m	28.3	1.76	m	20.6	1.59	m

		1.17	m					1.41	m
**16**	28.2	1.92	m	23.0	1.66	m	28.6	1.75	m
		1.32	m		1.36	m		1.35	m
**17**	50.4	1.48	m	55.7	1.26	m	56.1	1.22	m
**18**	15.7	0.69	s	12.0	0.63	s	12.8	0.82	s
**19**	19.1	0.98	s	16.3	0.95	s	18.2	0.88	s
**20**	36.2	1.38		40.4	2.04	m	39.7	2.02	m
**21**	18.6	0.91	d, *J*=6.4 Hz	21.1	1.04	d, *J*=6.7 Hz	20.9	1.00	d, *J*=6.6 Hz
**22**	36.3	1.43	m	135.6	5.18	dd, *J*=15.3, 7.3 Hz	135.2	5.14	dd, *J*=15.5, 8.4 Hz
		1.04	m						
**23**	24.9	2.03	m	131.9	5.21	dd, *J*=15.3, 7.3 Hz	132.3	5.22	dd, *J*=15.5, 7.7 Hz
		1.86	m						
**24**	125.2	5.10	m	42.8	1.85	m	42.7	1.85	m
**25**	130.9	-	-	33.1	1.47	m	33.0	1.47	m
**26**	17.6	1.60	s	19.9	0.84	d, *J*=6.8 Hz	19.6	0.82	d, *J*=6.8 Hz
**27**	25.7	1.68	s	19.6	0.82	d, *J*=6.8 Hz	19.9	0.83	d, *J*=6.8 Hz
**28**	27.9	1.00	s	17.6	0.92	d, *J*=6.8 Hz	17.5	0.91	d, *J*=6.8 Hz
**29**	15.4	0.81	s						
**30**	24.2	0.87	s						

Table S3. Complete ^{1}H and ^{13}C NMR resosnance assignments for compounds **3, 4 and 5** ($CDCl_3$)

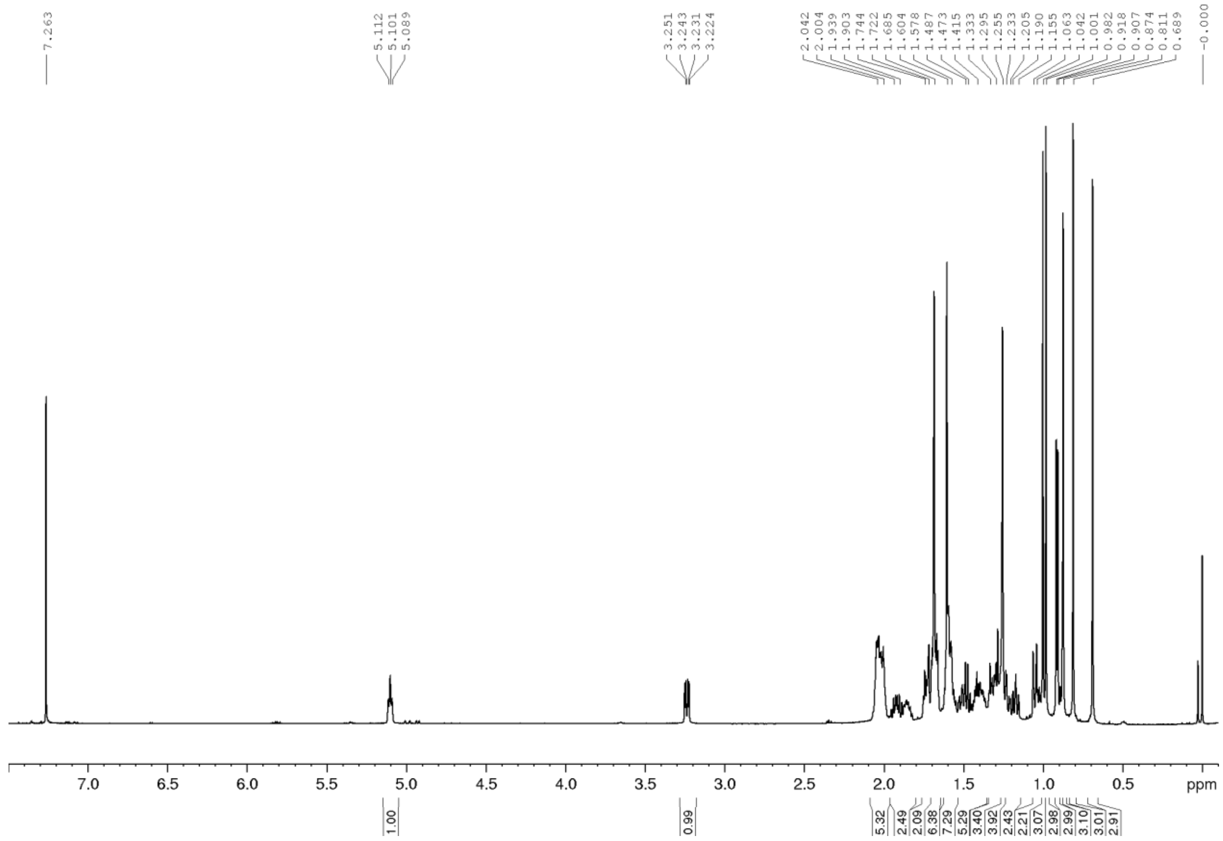

Figure S15.¹H NMR spectrum of compound **3** ($CDCl_3$)

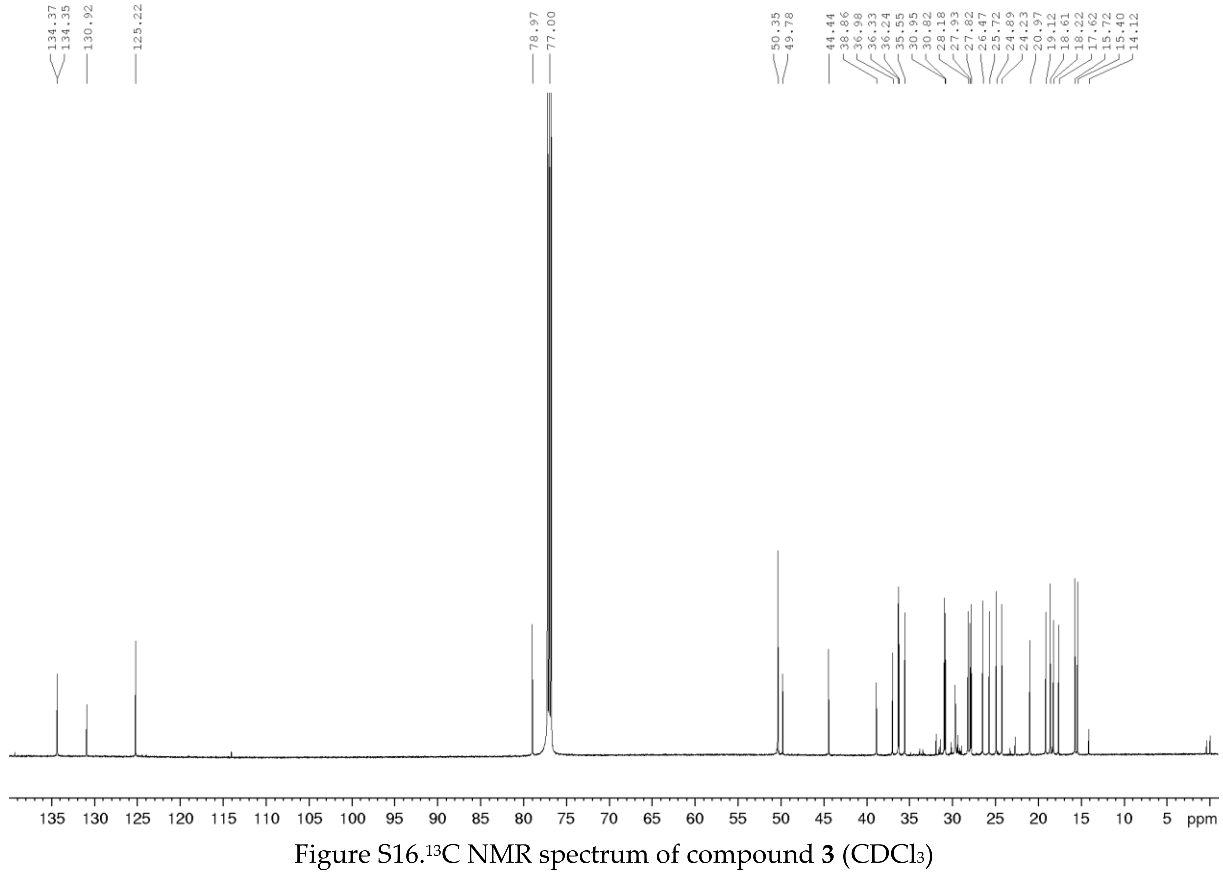

Figure S16.¹³C NMR spectrum of compound **3** ($CDCl_3$)

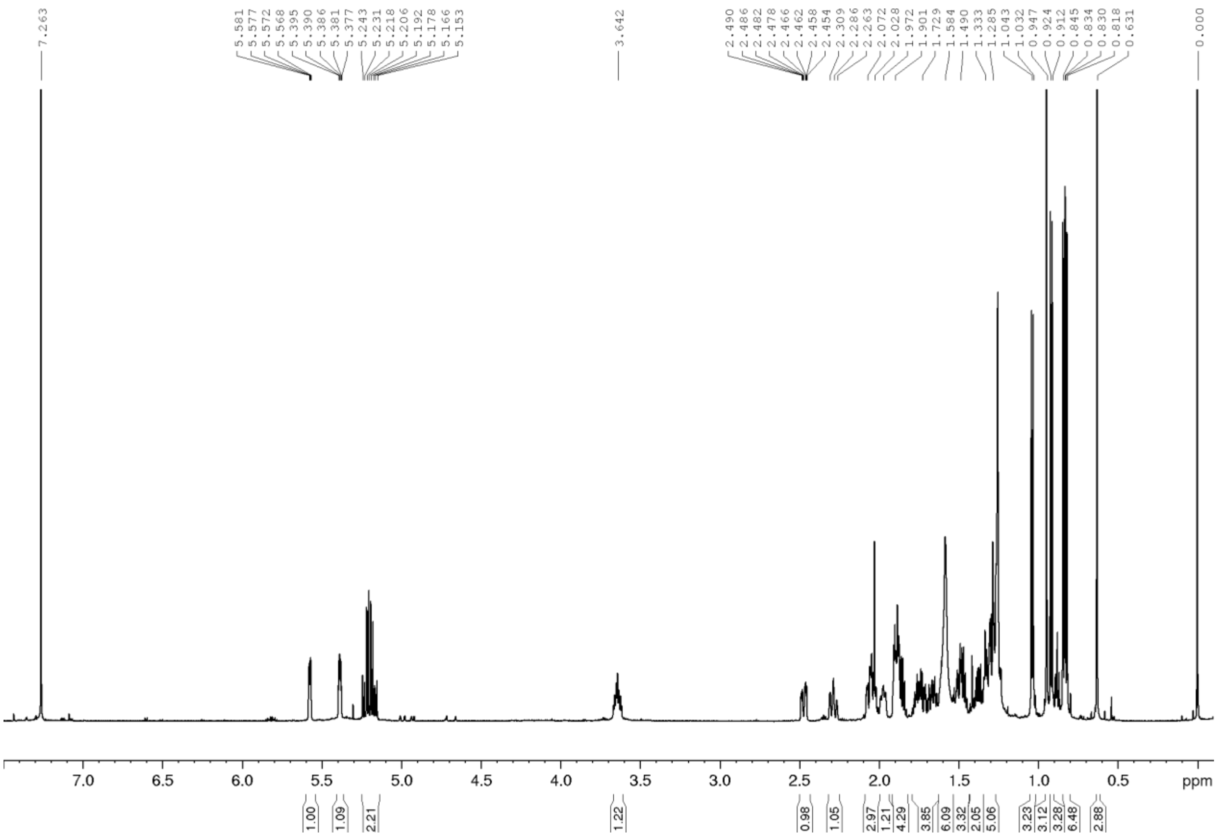

Figure S17.¹H NMR spectrum of compound **4** ($CDCl_3$)

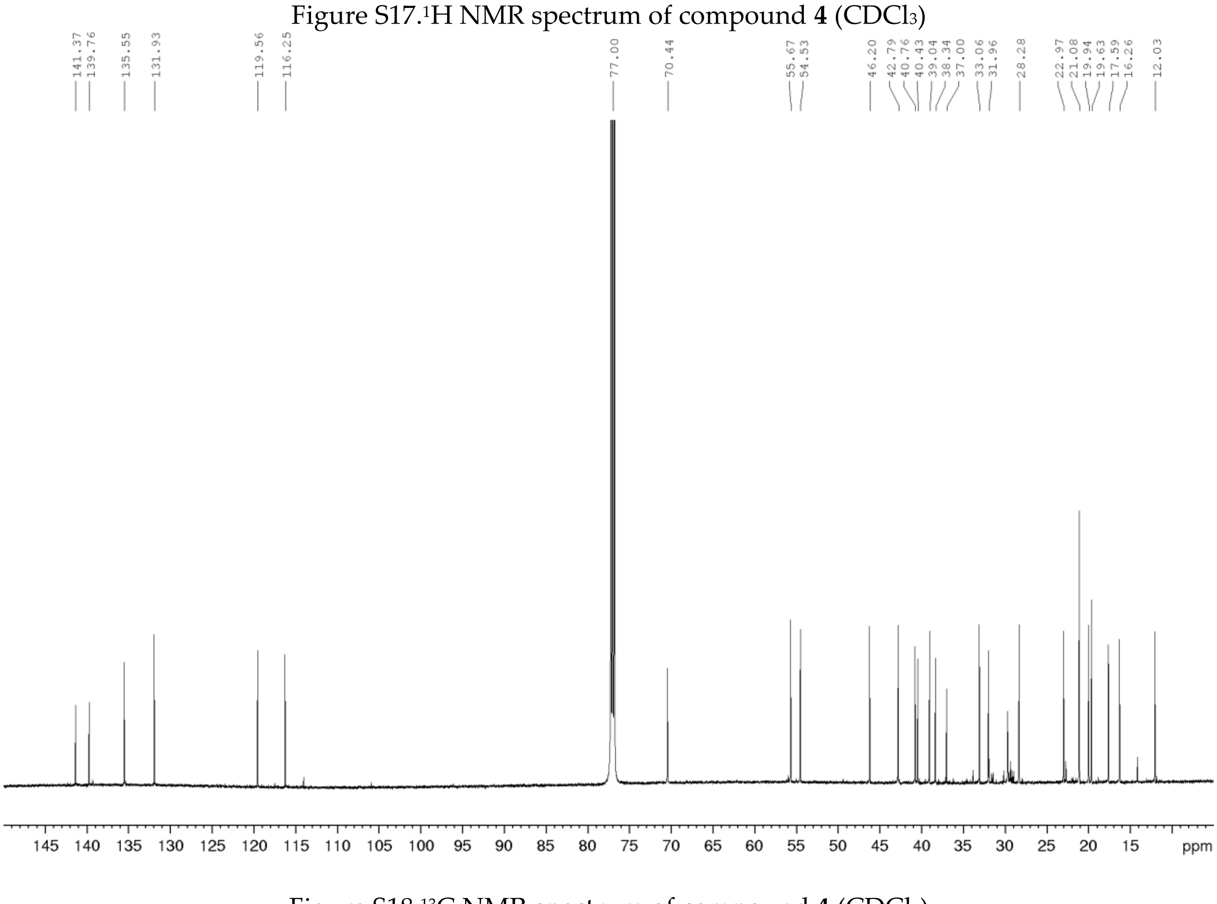

Figure S18.¹³C NMR spectrum of compound **4** ($CDCl_3$)

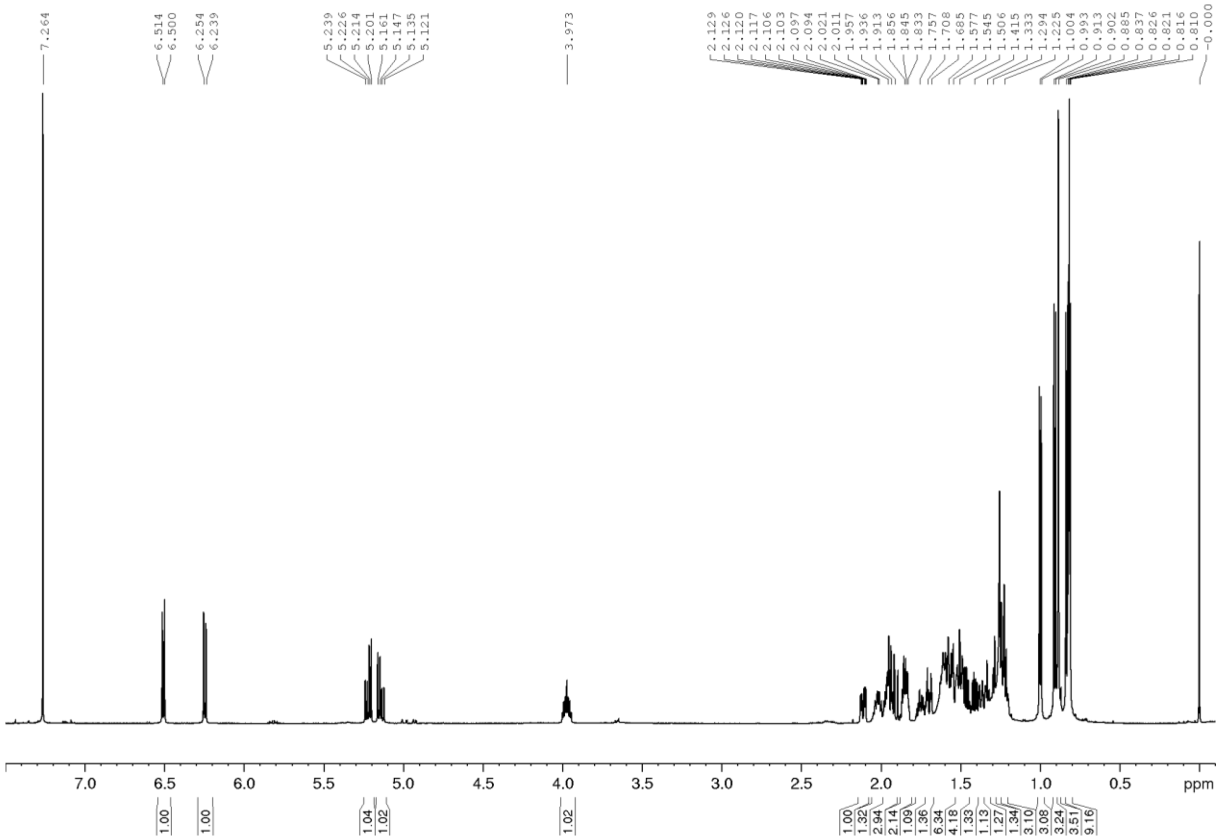

Figure S19.^{1}H NMR spectrum of compound **5** ($CDCl_3$)

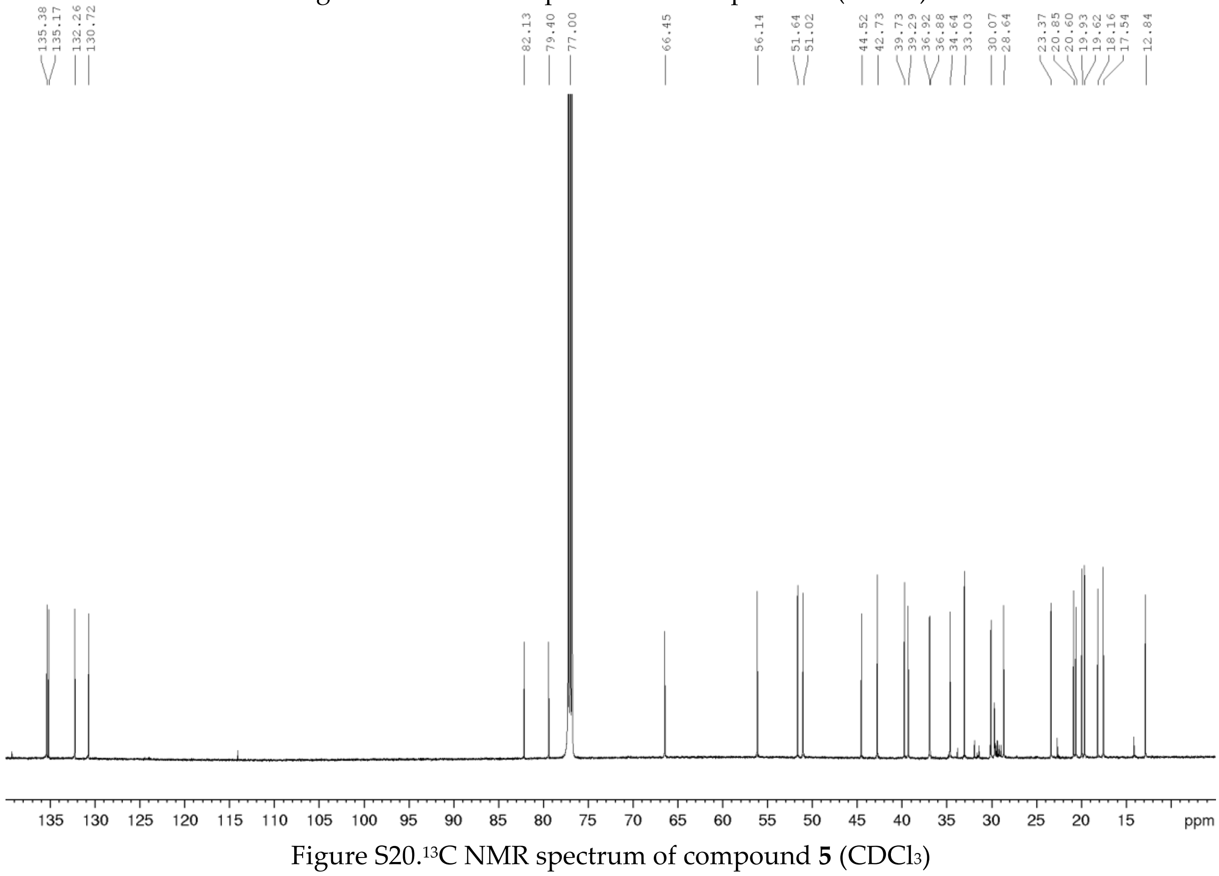

Figure S20.^{13}C NMR spectrum of compound **5** ($CDCl_3$)
